# The Impact of COVID-19 on Acute Surgical Admissions at the Sunshine Coast University Hospital

**DOI:** 10.7759/cureus.22644

**Published:** 2022-02-27

**Authors:** Brittany Long, David Grieve, Christopher Anstey

**Affiliations:** 1 Department of Surgery, Sunshine Coast University Hospital, Sunshine Coast, AUS; 2 School of Medicine, Griffith University, Sunshine Coast, AUS; 3 Department of Medical Education, Griffith University, Sunshine Coast, AUS

**Keywords:** general surgery, hospital resource utilization, hospitalized patients, coronavirus pandemic, covid-19

## Abstract

Purpose

It has been noted in international literature that acute surgical admissions and number of operations reduced as a result of coronavirus disease2019 (COVID-19). This study assesses the impact of the COVID-19 pandemic on the number of acute surgical admissions, operations, and length of stay (LoS) at the Sunshine Coast University Hospital (SCUH), Queensland, Australia.

Methodology

A retrospective study was conducted on patients admitted to the Acute Surgical Unit (ASU) during March and April for the years 2018, 2019, and 2020. Admission data for ASU patients in 2018 and 2019 were combined (pre-COVID) and compared with 2020 (COVID) to determine impact of the pandemic on presentations and procedures.

Results

ASU admissions reduced in 2020 (461 patients) compared with pre-COVID years (mean: 545 patients per year). There was an increase in the number (%) of operations performed in 2020, 175 patients (38%) compared with pre-COVID years, mean 158 patients (29%), p = 0.001. There was a significant decrease in the number (%) of functional presentations in 2020, 29 patients (6.3%) compared with pre-COVID years, mean 105 patients (9.6%), p = 0.04. LoS was not significantly different (52 hours vs. 54 hours, p = 0.11).

Conclusion

COVID-19 has reduced the absolute number of acute surgical admissions at SCUH. This effectively reduced triage workload. Contrary to the literature, this study did not demonstrate a reduction in the number of operations or change in LoS. These data could be used by health administrators to help with resource allocation during future pandemics.

## Introduction

In early March 2020, Australia reported its first death from coronavirus disease 2019 (COVID-19). Throughout the month of March, many more Australians tested positive for COVID-19. Educational institutions began to close, and airlines began suspending flights. By mid-March, Australia banned all international arrivals by non-citizens and residents. By the end of March, the Australian Prime Minister introduced lockdown and the closure of all non-essential services, and the Queensland Premier introduced tight social distancing restrictions. These lockdown laws had a profound effect on people’s movement and behaviors [1]. In 2021, these restrictions have continued, and lockdown rules are ever-changing.

Prior to the COVID-19 pandemic, there was a paucity of information in the literature with regard to surgical admission rates during pandemics. During the Ebola virus outbreak in West Africa, hospital admission rates reduced and surgical procedures reduced up to 50% [2-5]. COVID restrictions in Canada and Hong Kong resulted in a reduction in hospital admissions and surgical procedures [6,7].

Since the beginning of the pandemic, there have been multiple retrospective analyses on the effects of COVID-19 on different parts of the health sector. A study performed by O'Connell et al. in the Republic of Ireland assessed patients presenting to their emergency surgical service between March 1, 2020, and April 3, 2020, and compared it with the preceding three years. They noted a 42.8% reduction in the number of patients admitted and a 25.4% reduction in operations [8]. Another study by Callan et al. in the United Kingdom found a similar reduction in admissions, though the rate of operations remained the same as before [9]. A study performed by Moustakis et al. in South Africa compared surgical admissions in the weeks before lockdown and during the lockdown and found a reduction in both non-trauma and trauma admissions [10].

The COVID-19 pandemic has had a significant effect on health systems worldwide and in Australia [11]. While elective surgery was reduced to category 1 (defined as needing treatment within 30 days) cases only in both the public and private health systems on the Sunshine Coast, there was no change to emergency surgery or the rostering of the Acute Surgical Unit (ASU) in the Sunshine Coast Hospital and Health Service (SCHHS). Despite this, there has been an anecdotal decline in the number of acute surgical presentations across the SCHHS. The reason for this perceived reduction is unclear, and it is hypothesized that the COVID-19 pandemic should not affect acute surgical admissions on the Sunshine Coast, as it is presumed that acute surgical presentations should be independent of co-existing viral infection and community quarantine. The objective of this study is to assess the impact of the COVID-19 pandemic on the number of public acute surgical admissions and to document any variance in length of admission as well as operative versus non-operative management as a consequence of COVID-19. The SCHHS comprises the following public facilities: Sunshine Coast University Hospital (SCUH), Nambour General Hospital, Caloundra Hospital, Gympie Hospital, and Maleny Hospital. These facilities service the Sunshine Coast, Hinterland, and Gympie regions. The policy both pre-COVID and during COVID is for all acute surgical presentations at these peripheral hospitals to be transferred to SCUH ASU, therefore enabling us to capture all acute surgical admissions on the Sunshine Coast.

## Materials and methods

This retrospective clinical audit was performed at the SCUH. Ethics exemption was obtained from the Metro North Health Human Research Ethics Committee (HREC Reference: Project ID 64819 LNR/2020/QPCH/64819). Baseline patient demographic and clinical details were obtained via the electronic patient medical record (iEMR).

The study population comprised all patients who were admitted under the ASU at SCUH for the dates March 1 to April 30 for the years 2018, 2019, and 2020. The same time frame each year was used to minimize the effect of seasonal fluctuations. Patients were excluded from the study if the majority of their care took place at another facility, under another inpatient team, or if they were under the age of 18 years. Patients were further categorized by age, gender, length of stay, readmission within seven days, and disposition. Medical diagnosis was made based on the discharge summary or working diagnosis at the end of the admission if a discharge summary was not completed. Treatment was categorized as antibiotics, surgical, endoscopic, or radiological intervention. Endoscopic intervention included gastroscopy, colonoscopy, and endoscopic retrograde cholangiopancreatography (ERCP), while radiological intervention included percutaneous drainage, and embolization. Outcomes were categorized as discharged (treatment complete), discharged against medical advice, transferred to another facility, or died in hospital.

Within the diagnostic categories, the functional category represented patients with an impairment of normal bodily function without evidence of acute surgical pathology, trauma was defined as all patients who sustain a mechanism or display physiological features of trauma requiring admission, biliary pathology included cholelithiasis and all related complications, and malignancy was defined as presentations where symptoms were a direct consequence of cancer.

As the objective of the study was to analyze the effect of the global pandemic on acute surgical admissions, the data from 2018 and 2019 were combined as “pre-COVID” data and compared with 2020 as “COVID” data.

Where appropriate, summary statistics are presented as number (%) for binary and categorical data, mean (SD) for normally distributed continuous data, and median (IQR) for non-normal continuous data. The Shapiro-Wilk test was used to determine the normality or otherwise of the data. Further binary comparisons were performed using either the standard Student’s t-test for normally distributed continuous data or the Wilcoxon sign-rank test for non-normal continuous data or Fisher’s exact test for categorical data. Stata Version 15.0 (StataCorp, College Station, TX) was used throughout, and the level of significance was set at p < 0.05.

## Results

Demographic variables between the pre-COVID and COVID data are presented in Table 1. The only significant difference was an increase in male gender in 2020.

**Table 1:** Demographics for 2018/2019 and 2020 of acute surgical patient admissions. LoS and age were not normally distributed and are summarized as median (IQR). LoS, length of stay

Variable	Pre-COVID	COVID	p-Value
Age (years)	56 (38-73)	56 (37-71)	0.36
Male sex %	48%	54%	0.04
LoS (hours)	54 (25-97)	52 (33-96)	0.11
Patients per period	528/562	461	

The distribution of diagnoses from the pre-COVID and COVID data is presented in Table 2. In 2020, there was a statistically significant increase in the number of patients with appendicitis and malignancy. In 2020, there was a statistically significant decrease in the number of patients with a functional diagnosis and the number of patients with a hernia.

**Table 2:** Admission diagnosis for acute surgical admissions for 2018/2019 and 2020. I&D, incision and drainage; ERCP, endoscopic retrograde cholangiopancreatography; EUA, examination under anesthetic

Procedure	Number (%) of total admissions		p-Value
	Pre-COVID	COVID	
Appendicectomy	68 (6.2)	55 (11.9)	<0.001
Abscess I&D	69 (6.3)	44 (9.5)	0.03
ERCP	5 (0.5)	12 (2.6)	<0.001
No procedure	725 (66.4)	249 (54)	<0.001
Cholecystectomy	57 (5.2)	29 (6.3)	0.40
Bowel resection	31 (2.8)	13 (2.8)	1.00
Adhesiolysis	18 (1.7)	6 (1.3)	0.82
Upper endoscopy	11 (1.0)	4 (0.9)	1.00
Lower endoscopy	16 (1.5)	7 (1.6)	1.00
Percutaneous drainage	10 (0.9)	3 (0.7)	0.80
Radiological aspiration	12 (0.9)	7 (1.6)	0.30
Trauma laparotomy	7 (0.6)	3 (0.7)	1.00
Wound washout	24 (2.2)	11 (2.4)	0.85
Diagnostic laparoscopy	13 (1.3)	6 (1.3)	0.81
Embolisation	6 (0.6)	1 (0.2)	0.68
Hernia repair	14 (1.3)	6 (1.3)	1.00
EUA	2 (0.2)	4 (0.9)	0.07
Other	2 (0.2)	1 (0.2)	1.00
Total number of patients	1048	461	

Treatment interventions by category for pre-COVID and COVID patients have been presented in Table 3. There was a statistically significant increase in the percentage of patients who received antibiotics or who underwent surgery in 2020. The increase in endoscopic procedures from pre-COVID to COVID approached significance (p = 0.06).

**Table 3:** Treatment intervention by category for acute surgical admissions for 2018/2019 and 2020. *Not mutually exclusive

Interventions*	Number (%) of total admissions		p-Value
	Pre-COVID	COVID	
Antibiotics	488 (48)	263 (59)	<0.001
Surgical	295 (29)	169 (38)	<0.001
Endoscopic	33 (3.2)	24 (5.4)	0.06
Radiological	30 (2.9)	10 (2.3)	0.60

Further categorization of surgical, endoscopic, and radiological interventions has been presented in Table 4. In 2020, there were statistically significant increases in patients who underwent appendicectomy, abscess incision and drainage, and ERCP. There was a statistically significant decrease in the number of patients who did not undergo any procedure.

**Table 4:** Treatment Interventions for acute surgical admissions for 2018/2019 and 2020. I&D, incision and drainage; ERCP, endoscopic retrograde cholangiopancreatography; EUA, examination under anesthetic

Procedure	Number (%) of total admissions		p-Value
	Pre-COVID	COVID	
Appendicectomy	68 (6.2)	55 (11.9)	<0.001
Abscess I&D	69 (6.3)	44 (9.5)	0.03
ERCP	5 (0.5)	12 (2.6)	<0.001
No procedure	725 (66.4)	249 (54)	<0.001
Cholecystectomy	57 (5.2)	29 (6.3)	0.40
Bowel resection	31 (2.8)	13 (2.8)	1.00
Adhesiolysis	18 (1.7)	6 (1.3)	0.82
Upper endoscopy	11 (1.0)	4 (0.9)	1.00
Lower endoscopy	16 (1.5)	7 (1.6)	1.00
Percutaneous drainage	10 (0.9)	3 (0.7)	0.80
Radiological aspiration	12 (0.9)	7 (1.6)	0.30
Trauma laparotomy	7 (0.6)	3 (0.7)	1.00
Wound washout	24 (2.2)	11 (2.4)	0.85
Diagnostic laparoscopy	13 (1.3)	6 (1.3)	0.81
Embolization	6 (0.6)	1 (0.2)	0.68
Hernia repair	14 (1.3)	6 (1.3)	1.00
EUA	2 (0.2)	4 (0.9)	0.07
Other	2 (0.2)	1 (0.2)	1.00
Total number of patients	1048	461	

Analysis of the operative versus non-operative management of appendicitis and hernias has been presented in Table 5. Fisher’s exact test was used to compare operative rates for transferred patients, if it was assumed all, or none of the transferred patients underwent an operation.

**Table 5:** Rates of operative management for appendicitis and hernia between 2018/2019 and 2020 using Fisher’s exact test to account for patients transferred to other facilities. n = number of patients

	Appendicitis			Hernia		
	Pre-COVID	COVID	p-value	Pre-COVID	COVID	p-Value
Operation (n)	67	56		16	5	
No operation (n)	27	5		21	2	
Total number (n)	94	61		37	7	
Patients transferred to another facility (n)	23	0		3	0	
% Operated on	71%	92%	0.002	43%	71%	0.23
% Operated on if all transferred patients had an operation	94%	92%	0.75	51%	71%	0.43

## Discussion

The COVID-19 pandemic had a significant effect on Australian healthcare systems, including acute surgical patients. This retrospective analysis of acute surgical presentations during the COVID-19 pandemic and social isolation on the Sunshine Coast documented significant variation when compared with previous years. The number of admissions to the ASU for 2018 was 528 compared with 562 in 2019, while in 2020 the number of admissions was 461. The annual increase in total admissions in the pre-COVID years could be partly explained by the population increase on the Sunshine Coast from 356,823 to 361,870 (growth rate of 1.41%) [12]. Despite a population increase in 2020 to 367,180, there was an 18.34% decrease in the number of acute surgical admissions for 2020 compared with the average from the pre-COVID.

Analysis of admission diagnosis revealed that there was a significant decrease in the number of functional admissions to the ASU in 2020 when compared with previous years. In the pre-COVID data set, these admissions made up 9.6% of all acute surgical admissions, and during COVID, they only comprised 6.3% of the admissions. This decline could be explained by the public health lockdown initiatives, which may have deterred patients without pathology requiring surgical intervention from presenting to the hospital. Importantly, there was no significant decrease in patients with organic surgical pathologies in 2020 compared with previous years, with the exception of hernias. In the pre-COVID data set, the percentage of patients with a hernia diagnosis that proceeded to an operation was 37% compared with 86% for 2020. This suggests that during 2020, patients with uncomplicated hernias did not present to the hospital during lockdown.

The only pathologies with a statistically significant increase in 2020 were appendicitis (13.5% vs. 8.4%) and malignancy (1.7% vs. 0.6%). The percentage of patients with appendicitis who underwent an appendicectomy in 2018/2019 was 73% compared with 90% in 2020. In 2018, 17 patients with a diagnosis of appendicitis were transferred to Sunshine Coast University Private Hospital under a public/private contract. When it was assumed that all transferred patients underwent an appendicectomy, the rate of operatively managed appendicitis was 91%, indicating no change in rates of appendicectomy post-COVID. While there is current evidence to suggest that some cases of appendicitis can be managed conservatively with a similar efficacy to surgical intervention [13], this was not the policy of the SCHHS during the study years analyzed.

There was also a significant increase in the percentage of patients who underwent a procedure and/or received antibiotics in 2020 compared with previous years. This could be attributed to the increase in appendicitis and the decrease in patients with functional presentations and uncomplicated hernias.

There was no statistically significant difference between the pre-COVID and COVID populations with regard to the duration of stay and rates of readmission. This is an important note, as duration of stay and readmission rates are measures of morbidity. In contrast, a study in the United Kingdom found that the COVID-19 lockdown resulted in prolonged admissions and higher rates of complications [14]. Another study in New Zealand found that there were fewer acute surgical admissions, though there were increased rates of complications and length of stay [15].

It has been noted in the international literature that acute surgical admissions and number of operations reduced because of COVID-19. The SCUH noted a significant drop in acute surgical admissions; however, there was an increase in surgical operations and no significant decrease in patients with acute surgical pathology. This could be a consequence of the difference in the burden of COVID-19 between Australia and other countries. Although Australia had similar lockdown rules to other countries, our geographical isolation meant that there were significantly less cases of COVID-19 and that our health systems were not overwhelmed. There was no redirection of service provision in surgery to ICU and emergency at SCUH, and there was no reduced access to emergency theater nor changes in standard of operation (i.e. avoidance of laparoscopic surgery). This study, therefore, demonstrates that the reduction in acute surgical admissions is more likely a result of the public avoidance of hospitals rather than redistribution of workload and highlights the importance of the continued function of the ASU during future pandemics. It may also service as insight into resource allocation, possibly supporting the continuation of Categories 2 and 3 elective surgery throughout the pandemic.

While the study demonstrated that there was no significant change in the percentage of patients with acute surgical pathologies between 2020 and the pre-COVID years, this study is limited by the fact that the SCUH was only operational for four years prior to 2020, and an analysis of a longer time frame may improve the data. Further limitations include the minimal impact of COVID-19 on Australia in 2020 with low case numbers of COVID-19 and inconsistent and ever-changing lockdown rules at the start of the pandemic. Further analysis of the duration of symptoms and severity of disease at presentation may give an insight into whether the public health lockdown laws caused any detriment to patient outcomes by prolonging sickness prior to hospital presentation.

## Conclusions

This study demonstrated a decrease in the total number of acute surgical admissions, mainly in the functional presentation category. There was an increase in the percentage of patients who had surgery, but there was no significant change in the length of hospital stay. Further analysis into patient outcomes, such as time to surgery and post-operative complications, could be useful to assess other effects of the pandemic on patient care. This study provides valuable information regarding the possible trends to be anticipated in future global pandemics to help with resource allocation.
